# The First Step to Success

**Published:** 1914-03-21

**Authors:** 


### THE FIRST STEP TO SUCCESS.

To succeed, every doctor's wife who joins this effort to increase for her the purchasing power of every sovereign she may have available should carefully think out and put down on paper what proportion of her income, apart from that expended upon foodstuffs, she can properly make available for expenditure on co-operative principles. Local conditions may make it prudent to recognise that money spent locally can bring indirectly a good return apart from the actual articles purchased. But we are convinced that the doctor's wife can give full force to this consideration, and yet avail herself of the Association for the purchase and selection of many articles which will be outside and free from all local considerations. For instance, it is not an unusual practice for ladies resident in various parts of the country, when they find it inconvenient themselves to go to London or some other centre, to choose a trusted and experienced person to purchase for them articles and goods of which they enclose a full description with a remittance for an amount estimated to cover the cost of the goods in question. It would be a great advantage to many doctors' wives if they could arrange to have the material for their dresses or underclothing purchased on the co-operative principle and sent to them so that they would be enabled to have them made up under their personal direction in the district in which they reside. The same principle applies to large sections of articles such as those connected with sport and games, with the garden, the stable, and the many personal requirements for their husbands, themselves, and the children. These items or articles are merely suggested to enable every doctor's wife interested to think out what has been her expenditure in the last twelve months upon goods of the character indicated, and how far, if at all, the possession of an approved buyer in London, being one of the staff of the co-operation, would have saved her both time and expense, and secured an infinitely better result than she was able to arrive at without these facilities. We suggest the careful weighing and thoughtful examination of the points we have raised by every doctor's wife. **We invite correspondence, the making of inquiries, and the propounding of difficulties which may arise out of the study here invited, and are prepared to supply each correspondent with answers and practical help through experts who will constitute the staff, and who will always be at the disposal of every doctor's wife who joins the proposed Association.**

Time is the essence of success in a new departure like that we are discussing, and if success is to crown the desire to render substantial assistance to doctors' wives they must hasten in sufficient numbers to forward the coupon on page 678 with any correspondence and the formulation of any inquiries and difficulties which they may think it useful to submit at this stage. This is the first step to the practical establishment of the Association upon a basis best calculated to give the maximum of help and advantage to those doctors' wives who desire to become members of the Association. We therefore leave it to every doctor's wife to determine whether she will co-operate and energetically endeavour to add to the resources of herself and her household by this means.

